# Simultaneous loss of phospholipase C*δ*1 and phospholipase C*δ*3 causes cardiomyocyte apoptosis and cardiomyopathy

**DOI:** 10.1038/cddis.2014.181

**Published:** 2014-05-08

**Authors:** Y Nakamura, K Kanemaru, R Kojima, Y Hashimoto, T Marunouchi, N Oka, T Ogura, K Tanonaka, K Fukami

**Affiliations:** 1 Laboratory of Genome and Biosignals, Tokyo University of Pharmacy and Life Sciences, Hachioji, Tokyo, Japan; 2 CREST, JST, Kawaguchi, Saitama, Japan; 3 Department of Molecular and Cellular Pharmacology, Tokyo University of Pharmacy and Life Sciences, Hachioji, Tokyo, Japan

**Keywords:** PLC, cardiomyocyte, cardiac dilation, remodeling

## Abstract

Phospholipase C (PLC) is a key enzyme in phosphoinositide turnover. Among 13 PLC isozymes, PLC*δ*1 and PLC*δ*3 share high sequence homology and similar tissue distribution, and are expected to have functional redundancy in many tissues. We previously reported that the simultaneous loss of PLC*δ*1 and PLC*δ*3 caused embryonic lethality because of excessive apoptosis and impaired vascularization of the placenta. Prenatal death of PLC*δ*1/PLC*δ*3 double-knockout mice hampered our investigation of the roles of these genes in adult animals. Here, we generated PLC*δ*1/PLC*δ*3 double-knockout mice that expressed PLC*δ*1 in extra-embryonic tissues (cDKO mice) to escape embryonic lethality. The cDKO mice were born at the expected Mendelian ratio, which indicated that the simultaneous loss of PLC*δ*1 and PLC*δ*3 in the embryo proper did not impair embryonic development. However, half of the cDKO mice died prematurely. In addition, the surviving cDKO mice spontaneously showed cardiac abnormalities, such as increased heart weight/tibial length ratios, impaired cardiac function, cardiac fibrosis, dilation, and hypertrophy. Predating these abnormalities, excessive apoptosis of their cardiomyocytes was observed. In addition, siRNA-mediated simultaneous silencing of PLC*δ*1 and PLC*δ*3 increased apoptosis in differentiated-H9c2 cardiomyoblasts. Activation of Akt and protein kinase C (PKC) *θ* was impaired in the hearts of the cDKO mice. siRNA-mediated simultaneous silencing of PLC*δ*1 and PLC*δ*3 also decreased activated Akt and PKC*θ* in differentiated-H9c2 cardiomyoblasts. These results indicate that PLC*δ*1 and PLC*δ*3 are required for cardiomyocyte survival and normal cardiac function.

Dilated cardiomyopathy (DCM) is the most common type of non-ischemic cardiomyopathy, and is characterized by myocardial contractile dysfunction and cardiac diameter enlargement, which leads to heart failure. DCM is often accompanied by pathological remodeling, such as cardiac fibrosis and hypertrophy. In addition, apoptotic cardiomyocytes are observed in the hearts of humans with DCM.^1, 2, 3^ Interestingly, cardiomyocyte apoptosis was reported to be sufficient to induce adverse cardiac remodeling in an animal model.^4^

Phospholipase C (PLC) is a key enzyme in phosphoinositide turnover. PLC hydrolyzes phosphatidylinositol 4,5-bisphosphate (PIP_2_) to generate two second messengers, inositol 1,4,5-trisphosphate (IP_3_) and diacylglycerol.^5, 6^ There are 13 PLC isoforms in mammals, and they are divided into six types on the basis of sequence homology and activation mechanisms.^7, 8^ Among them, *δ*-type PLC has a relatively simple structure and is thought to be a primitive type of PLC isoform.^9^ We previously reported that the loss of PLC*δ*1 resulted in skin abnormalities in mice, such as hair loss, epidermal hyperplasia, and cytokine overproduction.^10, 11, 12, 13^ Disruption of the *PLCδ1* gene also protects mice from obesity by inhibiting lipid accumulation in adipose tissue.^14^ Regarding PLC*δ*3, siRNA-mediated silencing of PLC*δ*3 inhibits neuronal migration and neurite outgrowth.^15^ PLC*δ*1 and PLC*δ*3 are expressed in the heart, in addition to the skin, adipose, and neuronal tissues, and are expected to have critical roles in the cardiovascular system. The downstream effector of PLC, protein kinase C (PKC) has critical roles in cardiac structure and function. Several PKC isozymes are expressed in cardiomyocytes and regulate cardiac responses.^16^ Overexpression of PKC*α* in cultured cardiomyocytes induces hypertrophy.^17^ On the other hand, loss of PKC*α* prevents the transition from cardiac hypertrophy to cardiac failure.^18^ In addition, overexpression of PKC*β*_1_ leads to cardiac hypertrophy and sudden death.^19^ Thus, hyperactivation or overexpression of PKC*α* or PKC*β*_1_ is an inducible factor for cardiac hypertrophy and failure. In contrast, PKC*ɛ* protects the heart from apoptosis induced by ischemia and reperfusion injury,^20^ and PKC*θ* is required for cardiomyocyte survival and cardiac remodeling.^21^ The conventional PKC isozymes, PKC*α* and PKC*β*_I_ are activated by calcium and diacylglycerol, whereas the novel PKC isozymes, PKC*ɛ* and PKC*θ* require diacylglycerol but do not require calcium. Their distinct activation mechanisms may be involved in the difference in physiological functions between conventional and novel PKC isozymes. Both conventional and novel PKC isozymes are activated downstream of PLC. However, mice that lack either PLC*δ*1 or PLC*δ*3 do not show apparent cardiac abnormalities. As PLC*δ*1 and PLC*δ*3 share high sequence homology, one gene product likely compensates for the lack of the product of the other gene in mice. Unfortunately, the simultaneous loss of PLC*δ*1 and PLC*δ*3 results in embryonic lethality because of excessive apoptosis of placental trophoblasts and impaired vascularization of the placenta,^22^ which hampered our investigation into the role of the *PLCδ1* and *PLCδ3* genes in the cardiovascular systems in adult animals. In this study, we generated and analyzed mice that lack PLC*δ*1 and PLC*δ*3 with extra-embryonic PLC*δ*1 expression and found that the simultaneous loss of PLC*δ*1 and PLC*δ*3 results in DCM-like phenotypes that are associated with excessive apoptosis of cardiomyocytes.

## Results

### Extra-embryonic expression of PLC*δ*1 rescued embryonic lethality of *PLCδ1*^−/−^*PLCδ3*^−/*−*^ mice

**Figure 1:**
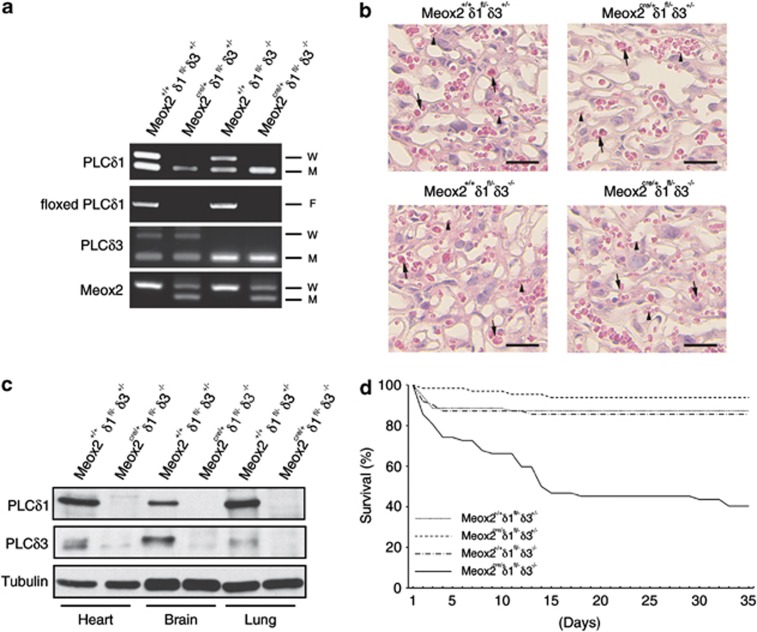
cDKO mice were born but died prematurely. (**a**) PCR analysis of genomic DNA from the tails of Meox2^+/+^PLC*δ*1^fl/−^PLC*δ*3^+/−^ (Meox2^+/+^*δ*1^fl/−^*δ*3^+/−^), Meox2^cre/+^PLC*δ*1^fl/−^PLCδ3^+/−^ (Meox2^cre/+^*δ*1^fl/−^*δ*3^+/−^), Meox2^+/+^PLCδ1^fl/−^PLCδ3^−/−^ (Meox2^+/+^*δ*1^fl/−^*δ*3^−/−^), and Meox2^cre/+^PLC*δ*1^fl/−^PLC*δ*3^−/−^ (Meox2^cre/+^*δ*1^fl/−^*δ*3^−/−^) mice. W, M, and F indicate PCR products from wild-type, mutant, and floxed alleles, respectively. (**b**) Hematoxylin and eosin staining of the labyrinth area of the placenta from Meox2^+/+^PLC*δ*1^fl/−^PLC*δ*3^+/−^ (Meox2^+/+^*δ*1^fl/−^*δ*3^+/−^), Meox2^cre/+^PLC*δ*1^fl/−^PLC*δ*3^+/−^ (Meox2^cre/+^*δ*1^fl/−^*δ*3^+/−^), Meox2^+/+^PLC*δ*1^fl/−^PLC*δ*3^−/−^ (Meox2^+/+^*δ*1^fl/−^*δ*3^−/−^), and Meox2^cre/+^PLC*δ*1^fl/−^PLC*δ*3^−/−^ (Meox2^cre/+^*δ*1^fl/−^*δ*3^−/−^) embryos at E13.5. The arrows indicate embryonal vessels and the arrowheads indicate maternal vessels. Scale bar, 25 *μ*m. (**c**) Immunoblotting of PLC*δ*1, PLC*δ*3, and tubulin in tissues from Meox2^+/+^PLC*δ*1^fl/−^PLC*δ*3^+/−^ (Meox2^+/+^*δ*1^fl/−^*δ*3^+/−^) and Meox2^cre/+^PLC*δ*1^fl/−^PLC*δ*3^−/−^ (Meox2^cre/+^*δ*1^fl/−^*δ*3^−/−^) mice. (**d**) Survival curves for Meox2^+/+^PLC*δ*1^fl/−^PLC*δ*3^+/−^ (Meox2^+/+^*δ*1^fl/*−*^*δ*3^+/*−*^), Meox2^cre/+^PLC*δ*1^fl/*−*^PLC*δ*3^+/*−*^ (Meox2^cre/+^*δ*1^fl/*−*^*δ*3^+/*−*^), Meox2^+/+^PLC*δ*1^fl/*−*^PLCδ3^*−*/*−*^ (Meox2^+/+^*δ*1^fl/−^*δ*3^−/−^), and Meox2^cre/+^PLC*δ*1^fl/−^PLC*δ*3^−/−^ (Meox2^cre/+^*δ*1^fl/−^*δ*3^−/−^) mice (Meox2^+/+^PLC*δ*1^fl/−^PLC*δ*3^+/−^, *n*=70; Meox2^+/+^PLC*δ*1^fl/−^PLC*δ*3^−/−^, *n*=64; Meox2^cre/+^PLC*δ*1^fl/−^PLC*δ*3^+/−^, *n*=62; and Meox2^cre/+^PLC*δ*1^fl/−^PLC*δ*3^−/−^cDKO, *n*=62)

We previously generated *PLCδ1*^−/−^*PLCδ3*^−/*−*^ mice (DKO mice) and found that they died *in utero* at embryonic day (E) 11.5–E13.5 because of excessive apoptosis of trophoblasts and placental vascular abnormalities.^22^ This embryonic lethality hampered our investigation into the roles of the *PLCδ1* and *PLCδ3* genes in adult animals. To avoid embryonic lethality, we generated mice that lacked PLC*δ*1 specifically in the embryo proper by using *Meox2*^*cre/+*^ mice, which express Cre recombinase in the embryo proper but not in extra-embryonic tissues, such as the placenta and yolk sac.^23^ Using these mice, we further generated *Meox2*^*Cre/*+^*PLCδ1*^fl/−^*PLCδ3*^−/*−*^ mice (cDKO mice) (Figure 1a). In contrast to DKO mice, the cDKO mice exhibited normal labyrinth architecture and vascularization in the placenta (Figure 1b). The cDKO mice were born at the expected Mendelian ratio (Table 1), which indicated that the simultaneous loss of PLC*δ*1 and PLC*δ*3 in the embryo proper did not result in embryonic lethality. We then confirmed that neither PLC*δ*1 nor PLC*δ*3 was expressed in the tissues of the adult cDKO mice, which indicated that mice that lacked both PLC*δ*1 and PLC*δ*3 were successfully obtained (Figure 1c). Loss of either PLC*δ*1 or PLC*δ*3 did not affect the postnatal survival rate. However, about half of the cDKO mice died in less than 2 weeks after birth (Figure 1d), which indicated that the simultaneous loss of PLC*δ*1 and PLC*δ*3 results in premature death.

### cDKO mice had hearts with abnormal morphologies and impaired function

**Figure 2:**
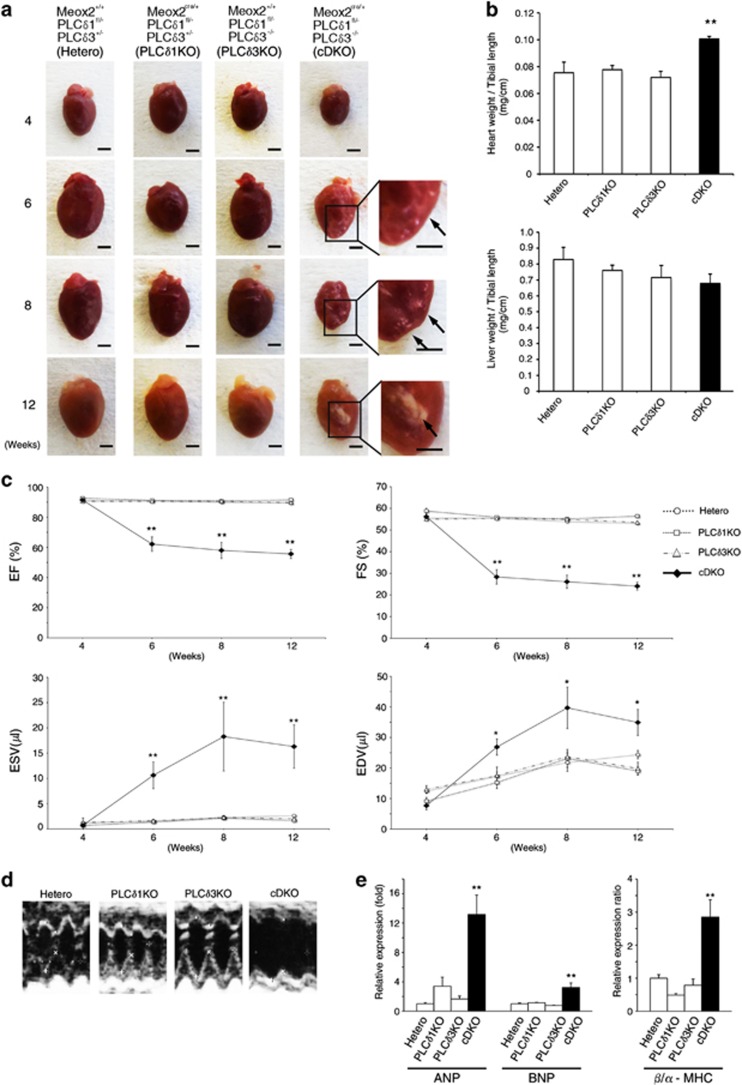
cDKO mice had hearts with abnormal morphologies and impaired functions. (**a**) Gross appearance of hearts from *Meox2*^*+/+*^*PLCδ1*^*fl/−*^*PLCδ3*^*+/−*^ (Hetero), *Meox2*^*cre/+*^*PLC*^*fl/−*^*PLCδ3*^*+/−*^ (PLC*δ*1KO), *Meox2*^*+/+*^*PLCδ1*^*fl/−*^*PLCδ3*^*−/−*^ (PLC*δ*3KO), and *Meox2*^*cre/+*^*PLCδ1*^*fl/−*^*PLCδ3*^*−/−*^ (cDKO) mice at 4, 6, 8, and 12 weeks of age. Scale bar, 2 mm. The boxed areas in the cDKO hearts at 6, 8, and 12 weeks are magnified in the right panels. The arrows indicate white foci. (**b**) Heart (upper panel) or liver (lower panel) weight-to-tibial length ratios of *Meox2*^*+/+*^*PLCδ1*^*fl/−*^*PLCδ3*^*+/−*^ (hetero), *Meox2*^*cre/+*^*PLC*^*fl/−*^*PLCδ3*^*+/−*^ (PLC*δ*1KO), *Meox2*^*+/+*^*PLCδ1*^*fl/−*^*PLCδ3−/−* (PLC*δ*3KO), and *Meox2*^*cre/+*^*PLCδ1*^*fl/−*^*PLCδ3*^*−/−*^ (cDKO) mice. Mean+S.E.M. (hetero, *n*=3; PLC*δ*1KO, *n*=6; PLC*δ*3KO, *n*=6; and cDKO, *n*=5). (**c**) Quantitative data of echocardiographic measurements. Echocardiograms were measured in *Meox2*^*+/+*^*PLCδ1*^*fl/−*^*PLCδ3*^*+/−*^ (hetero), *Meox2*^*cre/+*^*PLC*^*fl/−*^*PLCδ3*^*+/−*^ (PLC*δ*1KO), *Meox2*^*+/+*^*PLCδ1*^*fl/−*^*PLCδ3*^*−/−*^ (PLC*δ*3KO), and *Meox2*^*cre/+*^*PLCδ1*^*fl/−*^*PLCδ3*^*−/−*^ (cDKO) mice at 4, 6, 8, and 12 weeks of age. Mean±S.E.M. (hetero, *n*=6; PLC*δ*1KO, *n*=6; PLC*δ*3KO, *n*=7; and cDKO, *n*=5 at each time point). EF, ejection fraction; FS, fraction shortening; ESV, end-systolic volume; and EDV, end-diastolic volume. (**d**) Representative M-mode echocardiograms of *Meox2*^*+/+*^*PLCδ1*^*fl/−*^*PLCδ3*^*+/−*^ (hetero), *Meox2*^*cre/+*^*PLC*^*fl/−*^*PLCδ3*^*+/−*^ (PLC*δ*1KO), *Meox2*^*+/+*^*PLCδ1*^*fl/−*^*PLCδ3*^*−/−*^ (PLC*δ*3KO), and *Meox2*^*cre/+*^*PLCδ1*^*fl/−*^*PLCδ3*^*−/−*^ (cDKO) mice at 8 weeks of age. (**e**) mRNA expression or ratios of cardiac failure markers in the hearts of *Meox2*^*+/+*^*PLCδ1*^*fl/−*^*PLCδ3*^*+/−*^ (hetero), *Meox2*^*cre/+*^*PLC*^*fl/−*^*PLCδ3*^*+/−*^ (PLC*δ*1KO), *Meox2*^*+/+*^*PLCδ1*^*fl/−*^*PLCδ3*^*−/−*^ (PLC*δ*3KO), and *Meox2*^*cre/+*^*PLCδ1*^*fl/−*^*PLCδ3*^*−/−*^ (cDKO) mice at 8 weeks of age. The results are listed in arbitrary units (expression or ratio in hetero=1). Mean+S.E.M. (hetero, *n*=6; PLC*δ*1KO, *n*=3; PLC*δ*3KO, *n*=3; and cDKO, *n*=4). Statistical significance was assessed using Tukey–Kramer's *post hoc* test. *P<0.05 and **P<0.01

We next analyzed cDKO mice that survived the first 4 weeks. Although surviving cDKO mice had a normal life span, they had smaller body sizes than did the mice that lacked either PLC*δ*1 or PLC*δ*3. Morphological analysis revealed that *Meox2*^*+/+*^*PLCδ1*^*fl/−*^*PLCδ3*^*+/−*^, *Meox2*^*cre/+*^*PLCδ1*^*fl/−*^*PLCδ3*^*+/−*^, and *Meox2*^*+/+*^*PLCδ1*^*fl/−*^*PLCδ3−/−* mice had normal-shaped hearts, whereas the cDKO mice had irregular-shaped hearts with white foci after 6 weeks of age (arrows in Figure 2a). In addition, the heart weight/tibial length ratio was significantly increased for the cDKO mice (Figure 2b). Although heart weights were not significantly increased, cDKO mice have smaller body and their tibial length was shorter than that of control mice, resulting in increase in heart weight/tibial length ratio. This increase in the ratio was not observed in other organs, such as liver, indicating that the increase in the heart weight/tibial length ratio was specific. Because the cDKO hearts showed abnormal morphologies, we examined their cardiac function by performing echocardiography (Figures 2c and d). The ejection fraction (EF) and fractional shortening (FS) are echocardiographic indicators of overall cardiac function. There were no significant differences in the EF and FS among all genotypes at 4 weeks of age (Figure 2c). After 6 weeks, both the FS and EF were significantly decreased in the cDKO mice (Figure 2c). These results suggest that the cDKO mice had impaired cardiac function, and that this impairment occurred between 4 and 6 weeks of age, which was concomitant with their morphological abnormalities (Figure 2a). In addition to the EF and FS, the end-systolic volume and end-diastolic volume were increased in the cDKO hearts (Figure 2c), which indicated that the cDKO mice showed DCM-like phenotypes. Because the induction of natriuretic peptides ANP and BNP is a marker of cardiac failure, we measured the mRNA levels of ANP and BNP in the cDKO hearts. At 8 weeks of age, both the ANP and BNP were upregulated in the cDKO hearts (Figure 2e). The cDKO hearts also exhibited the *α*-to-*β* isoform switch of myosin heavy chain (MHC) and an increased ratio of *β*-MHC/*α*-MHC expression, which are typical features of heart failure at 8 weeks of age (Figure 2e). Thus, the cDKO hearts showed cardiac dysfunction and upregulation of cardiac failure markers. It is possible that the cardiac abnormalities in the cDKO mice were attributable to right ventricle cardiomyopathy. Therefore, we examined whether abnormal structure of the right ventricle and subsequent structural changes in the lungs were observed in cDKO mice. There were no apparent changes in the structure of the lungs or the right ventricle in cDKO mice, suggesting that the cardiac phenotypes were not caused by right ventricle cardiomyopathy (Supplementary Figure S1). In our mating strategy, *Meox2*^*+/+*^*PLCδ1*^*fl/−*^*PLCδ3*^*+/−*^, *Meox2*^*cre/+*^*PLCδ1*^*fl/−*^*PLCδ3+/−*, and *Meox2*^*+/+*^*PLCδ1*^*fl/−*^*PLCδ3*^*−/−*^ mice were obtained as littermates of cDKO (*Meox2*^*cre/+*^*PLCδ1*^*fl/−*^*PLCδ3−/−*) mice (Table 1). Any cardiac abnormalities that were observed in the cDKO mice were not observed in the littermates with other genotypes. Therefore, the littermates of the cDKO mice were used as a control in subsequent experiments, irrespective of their genotypes.

### Simultaneous loss of PLC*δ*1 and PLC*δ*3 caused cardiac fibrosis and hypertrophy

**Figure 3:**
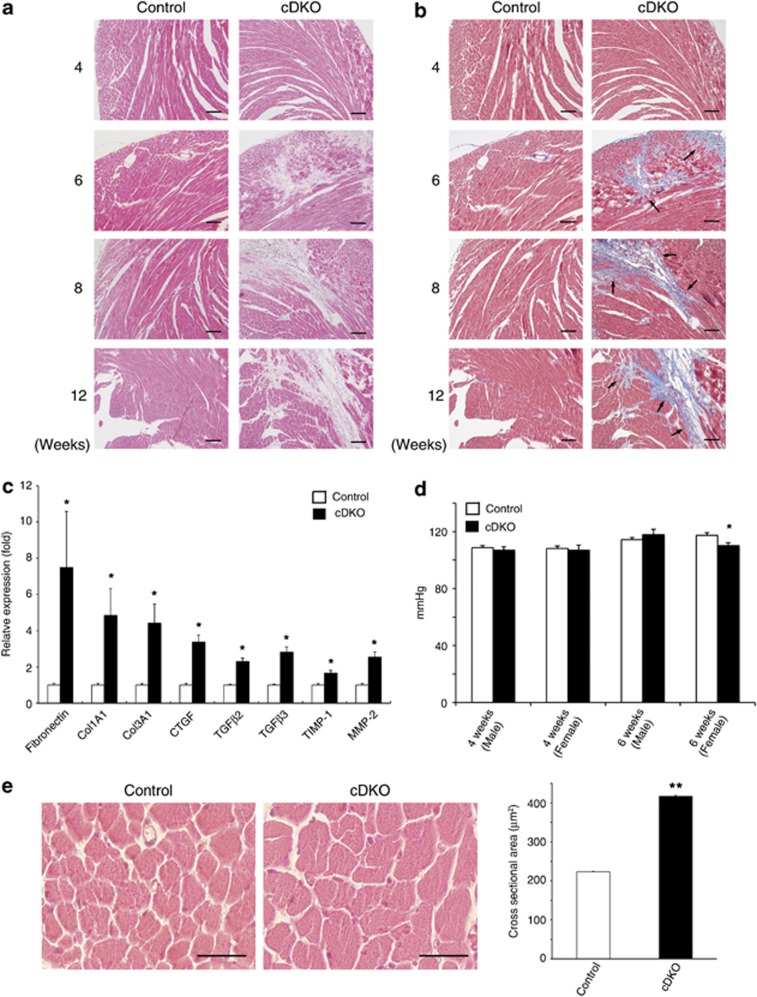
cDKO mice showed cardiac fibrosis. (**a**, **b**) Hematoxylin and eosin (**a**) and Masson trichrome (**b**)-stained sections from the left ventricles of control (*Meox2*^*+/+*^*PLCδ1*^*fl/−*^*PLCδ3*^*+/−*^) and cDKO mice at 4, 6, 8, and 12 weeks of age. The arrows indicate fibrotic areas (blue). Scale bar, 100 *μ*m. (**c**) mRNA expression of cardiac fibrosis markers in the hearts of cDKO mice at 8 weeks of age. The results are listed in arbitrary units (expression in control mice=1). Mean+S.E.M. (control, *n*=12; cDKO, *n*=4). (**d**) Systolic, diastolic blood pressure of 4- or 6-week-old control and cDKO mice. Mean+S.E.M. (4 weeks, male: control, *n*=9; cDKO, *n*=4; 4 weeks, female: control, *n*=9; cDKO, *n*=3; 6 weeks, male: control, *n*=10; cDKO, *n*=4; 6 weeks, female, control, *n*=11; and cDKO, *n*=4). (**e**) Hematoxylin and eosin-stained sections from left ventricles of control (*Meox2*^*cre/+*^*PLCδ1*^*fl/−*^*PLCδ3*^*+/−*^), and cDKO mice at 12 weeks of age. Scale bar, 50 *μ*m. Quantitative data of myocyte cross-sectional areas are also shown. Mean+S.E.M. 100 cells per section. (Control, *n*=3 and cDKO, *n*=3). Statistical significance was assessed using Student's *t*-test. *P<0.05 and **P<0.01

Because the cDKO mice showed cardiac dysfunction, we examined detailed histological structures of their hearts. Hematoxylin and eosin (HE) staining revealed that the cDKO hearts had fibrotic lesions (Figure 3a). Although these fibrotic lesions were not observed at 4 weeks of age, fibrosis was observed in the ventricular walls after 6 weeks of age (Figure 3a). We also stained collagen fibers by performing trichrome staining and found that the ventricular walls exhibited strong positive staining after 6 weeks of age (means±S.E.M. fibrosis*-*to*-*total ventricular area ratios, 0.0074±0.00096 in 6-week-old *Meox2*^*+/+*^*PLCδ1*^*fl/−*^*PLCδ3*^*+/−*^, 0.087±0.0095 in 6-week-old cDKO, 0.0071±0.0011 in 12-week-old *Meox2*^*+/+*^*PLCδ1fl/−PLCδ3*^*+/−*^, and 0.094±0.0075 in 12-week-old cDKO mice; *n*=3, for each) (Figure 3b). We further confirmed the occurrence of cardiac fibrosis at the molecular level by assessing the expression of fibrosis-related genes. Real-time reverse transcriptase PCR (RT-PCR) revealed that extracellular matrix components, such as fibronectin and collagen (Col1A1 and Col3A1), were upregulated in the cDKO hearts (Figure 3c and Supplementary Table S1). We also observed the upregulation of pro-fibrotic factors, such as connective tissue growth factor (CTGF), transforming growth factor (TGF)*β*2, and TGF*β*3 in the cDKO hearts (Figure 3c and Supplementary Table S1). The markers for cardiac remodeling that were associated with cardiac fibrosis, such as tissue inhibitors of metalloproteinase 1 (TIMP-1) and matrix metalloproteinase 2 (MMP-2), were also upregulated in the cDKO hearts (Figure 3c and Supplementary Table S1). These results indicate that the cDKO hearts showed cardiac fibrosis at both the histological and molecular levels. Because elevated blood pressure may be a cause for cardiac failure and fibrosis, we measured the blood pressures of the cDKO mice. The cDKO mice did not show remarkable changes in their systolic blood pressure when compared with the control mice (Figure 3d and Supplementary Table S2), which indicated that their cardiac fibrosis was not caused by hypertension. Apart from cardiac fibrosis, the cDKO cardiomyocytes were hypertrophic. Although the cell size remained unchanged at 2 and 4 weeks of age, the cardiomyocyte cross-sectional areas increased at 12 weeks of age (Figure 3e). Thus, cDKO hearts showed signs of pathological remodeling, such as cardiac fibrosis and hypertrophy, which is often associated with DCM.

### Simultaneous depletion of PLC*δ*1 and PLC*δ*3 caused cardiomyocyte apoptosis

**Figure 4:**
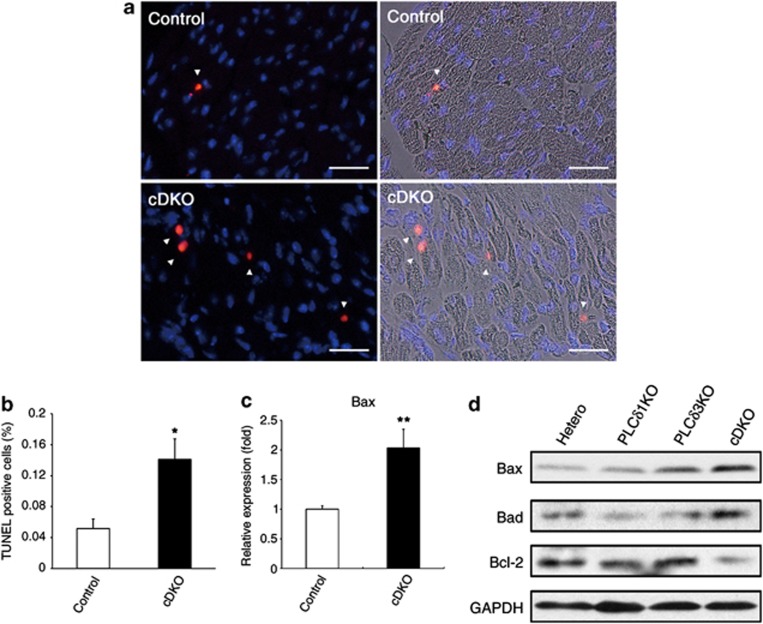
cDKO mice showed excessive apoptosis of cardiomyocytes. (**a**) TUNEL staining (red) of hearts from 4-week-old control (*Meox2*^*+/+*^*PLCδ1*^*fl/−*^*PLCδ3*^*+/−*^) and cDKO mice. Hoechst (blue) was used for nuclear staining. The arrowheads indicate TUNEL-positive cells. The right-hand panels are overlay images of bright field and fluorescent images. Scale bar, 50 μm. (**b**) Population of TUNEL-positive cells in the hearts of 4-week-old control (*Meox2*^*+/+*^*PLCδ1*^*fl/−*^*PLCδ3*^*+/−*^) and cDKO mice. Mean+S.E.M. (control, *n*=4; cDKO, *n*=3). (**c**) mRNA expression of the proapoptotic gene *Bax* in the hearts of control (*Meox2*^*+/+*^*PLCδ1*^*fl/−*^*PLCδ3*^*−/−*^, *n*=2; *Meox2*^*cre/+*^*PLC*^*fl/−*^*PLCδ3*^*+/−*^, *n*=2; *Meox2*^*+/+*^*PLCδ1*^*fl/−*^*PLCδ3*^*+/−*^ *n*=3) and cDKO mice at 4 weeks of age. The results are listed in arbitrary units (expression in control mice=1). Mean+S.E.M. (control, *n*=7; cDKO, *n*=4). Statistical significance was assessed using Student's *t*-test. *P<0.05 and **P<0.01. (**d**) Immunoblotting of Bax, Bad, Bcl-2, and GAPDH in *Meox2*^*+/+*^*PLCδ1*^*fl/−*^*PLCδ3*^*+/−*^ (hetero), *Meox2*^*cre/+*^*PLC*^*fl/−*^*PLCδ3*^*+/−*^ (PLC*δ*1KO), *Meox2*^*+/+*^*PLCδ1*^*fl/−*^*PLCδ3*^*−/−*^ (PLC*δ*3KO), and *Meox2*^*cre/+*^*PLCδ1*^*fl/−*^*PLCδ3*^*−/−*^ (cDKO) hearts at 8 weeks of age

Because apoptotic loss of cardiomyocytes leads to cardiac dysfunction and remodeling,^4^ we determined the number of apoptotic cells in the heart ventricles of cDKO mice that were 4 weeks of age before the onset of these cardiac abnormalities. A terminal transferase dUTP nick end labeling (TUNEL) assay revealed that apoptotic cardiomyocytes were easily found in the cDKO ventricles, whereas apoptotic cardiomyocytes were rarely observed in control ventricles (means±S.E.M. for TUNEL-positive cells, 0.051±0.021% in *Meox2*^*+/+*^*PLCδ1fl/−PLCδ3*^*+/−*^, 0.050±0.0031% in *Meox2*^*cre/+*^*PLCδ1*^*fl/−*^*PLCδ3*^*+/−*^, 0.057±0.0047% in *Meox2*^*+/+*^*PLCδ1*^*fl/−*^*PLCδ3*^*−/−*^, and 0.14±0.046% in cDKO mice; *n*=3 for each) (Figures 4a and b). There were neither apparent structural abnormalities nor an increase in the number of TUNEL-positive cells in the hearts of E17.5 embryos, compared with those in the hearts of control embryos, strongly suggesting that PLC*δ*1 and PLC*δ*3 were not required for cardiac protection during normal development (Supplementary Figure S2). We also confirmed that mRNA expression of the proapoptotic gene *Bax* was significantly increased in the cDKO hearts at 4 weeks of age (Figure 4c). In addition, western blotting showed that levels of proapoptotic proteins, Bax and Bad were increased in cDKO hearts, whereas that of antiapoptotic protein, Bcl-2, were decreased in the cDKO hearts (Figure 4d). Thus, the cDKO mice showed excessive apoptosis of cardiomyocytes.

**Figure 5:**
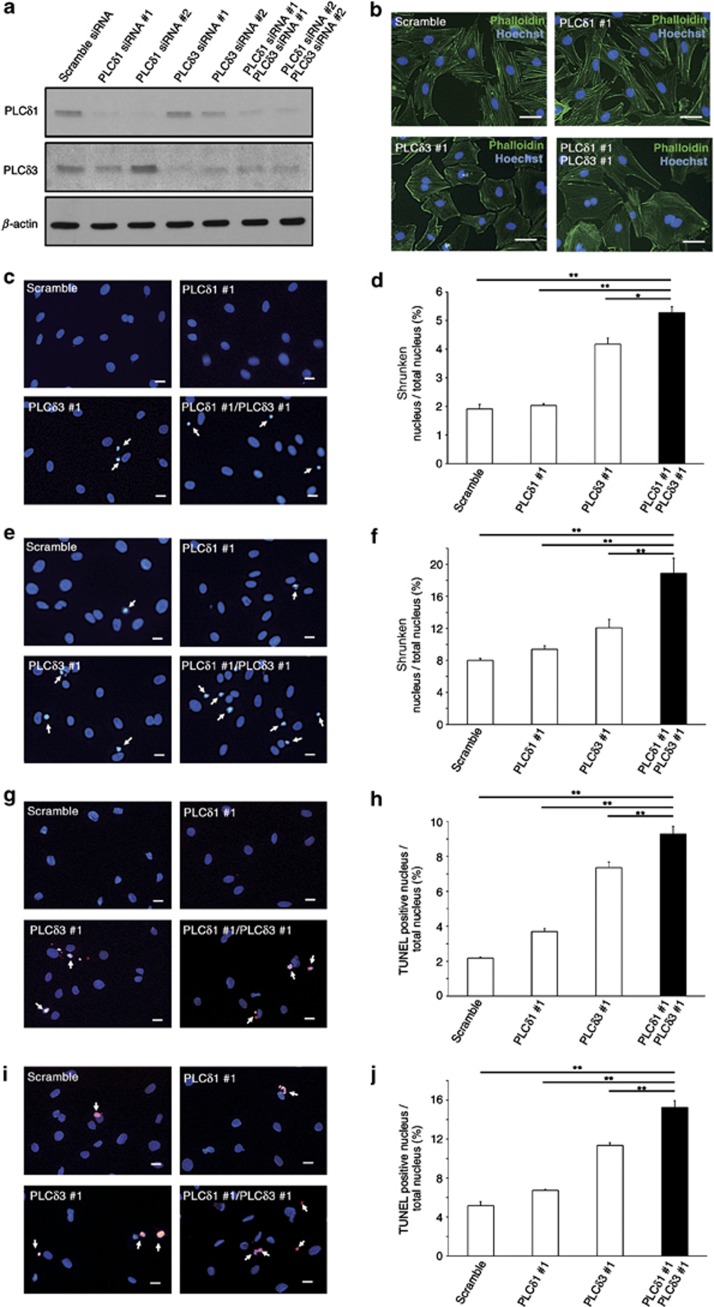
siRNA-mediated simultaneous silencing of PLC*δ*1 and PLC*δ*3 increased apoptosis in a cardiomyocyte cell line. (**a**) Immunoblotting of PLC*δ*1, PLC*δ*3, and *β*-actin in differentiated-H9c2 cells that were transfected with scrambled, PLC*δ*1-targeting (#1 and #2), or PLC*δ*3-targeting (#1 and #2) siRNA. (**b**) Morphologies of differentiated-H9c2 cells with the indicated siRNA. The cells were stained with phalloidin (green) and Hoechst (blue). Scale bar, 25 *μ*m. (**c**–**f**) Population of H9c2 cells with shrunken nuclei under normal culture conditions (**c** and **d**) or oxidative stress conditions (**e** and **f**). The arrows indicate shrunken nuclei. Scale bar, 25 *μ*m. Mean+S.E.M. (**g**–**j**) Population of H9c2 cells with TUNEL-positive nuclei under normal culture conditions (**g** and **h**) or oxidative stress conditions (**i** and **j**). The arrows indicate TUNEL-positive nuclei. Scale bar, 25 *μ*m. Mean+S.E.M. (**b**–**j**) The data were obtained with scrambled, PLC*δ*1-targeting (#1), and/or PLC*δ*3-targeting (#1) siRNA. Similar results were obtained with scrambled, PLC*δ*1-targeting (#2), and/or PLC*δ*3-targeting (#2) siRNA. Statistical significance was assessed using Tukey–Kramer's *post hoc* test. *P<0.05 and **P<0.01

We further carried out siRNA-mediated simultaneous knockdown of PLC*δ*1 and PLC*δ*3 in H9c2 rat cardiomyoblasts with two distinct siRNA that target PLC*δ*1 and PLC*δ*3, and induced the differentiation of H9c2 cells into cardiomyocytes with low-serum differentiation-promoting medium. Western blotting revealed that the amounts of PLC*δ*1 and PLC*δ*3 proteins were decreased in the differentiated-H9c2 cells by the introduction of the siRNA (Figure 5a), which indicated the effective knockdown of PLC*δ*1 and PLC*δ*3. The simultaneous knockdown of PLC*δ*1 and PLC*δ*3 resulted in cell spreading (Figure 5b), which suggests that PLC*δ*1 and PLC*δ*3 regulate the morphology of cardiomyocytes. Unexpectedly, similar morphological changes were observed by the single knockdown of PLC*δ*3 (Figure 5b), which suggests that PLC*δ*3 downregulation is, in contrast to *in vivo* case, sufficient for the induction of abnormal morphology of cardiomyocytes *in vitro*. In addition to morphological changes, simultaneous downregulation of PLC*δ*1 and PLC*δ*3 induces nuclear shrinking, which is a typical feature of apoptosis under normal culture conditions (Figures 5c and d) and oxidative stress conditions (Figures 5e and f). We further confirmed the apoptosis of cardiomyocytes by TUNEL staining and found that simultaneous silencing of PLC*δ*1 and PLC*δ*3 increased the number of TUNEL-positive cells under normal culture conditions (Figures 5g and h) and oxidative stress conditions (Figures 5i and j). Although the extent was milder than the extent in double-knockdown cells, single knockdown of PLC*δ*3 also induced apoptosis. Taken together, these findings suggest that simultaneous depletion of PLC*δ*1 and PLC*δ*3 increased the apoptosis of cardiomyocytes in a cell-intrinsic manner.

### Simultaneous depletion of PLC*δ*1 and PLC*δ*3 impaired the activation of PKC*θ*

**Figure 6:**
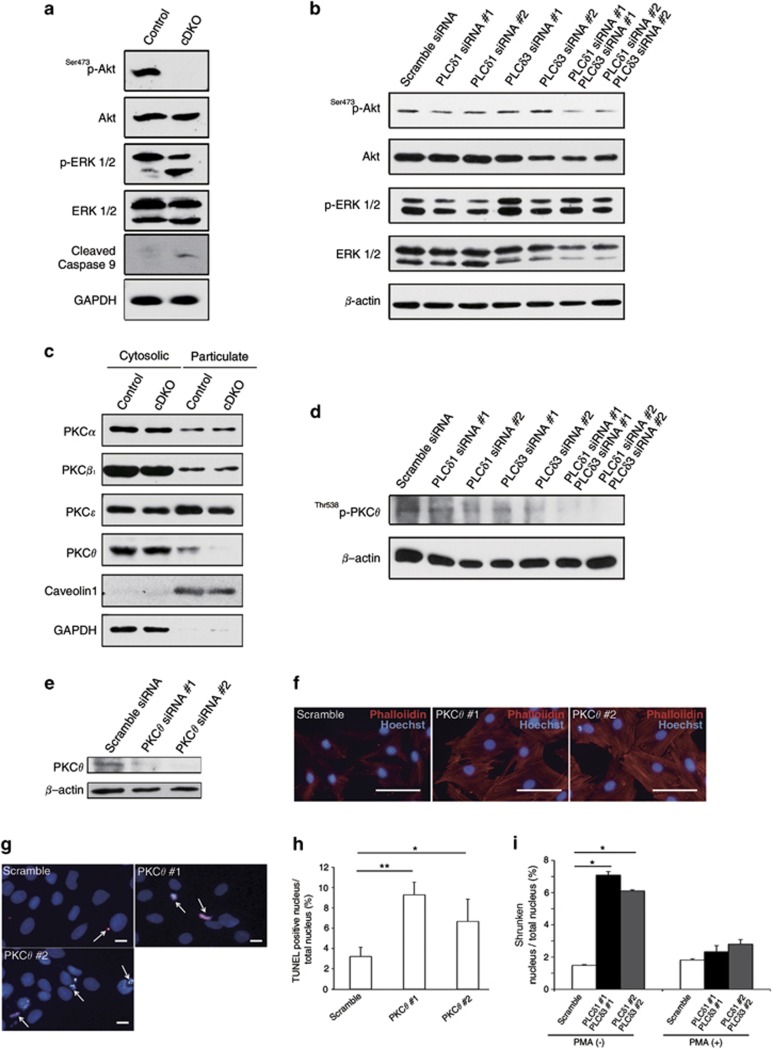
Simultaneous depletion of PLC*δ*1 and PLC*δ*3 impaired activation of Akt and PKC*θ*. (**a**) Immunoblotting of the phosphorylated form of Akt (^Ser473^p-Akt), total Akt (Akt), phosphorylated form of ERK1/2 (p-ERK1/2), total ERK1/2 (ERK1/2), cleaved Caspase 9, and GAPDH in control (*Meox2*^*+/+*^*PLCδ1*^*fl/−*^*PLCδ3*^*+/−*^) or cDKO hearts at 8 weeks of age. (**b**) Immunoblotting of the phosphorylated form of Akt (^Ser473^p-Akt), total Akt (Akt), phosphorylated form of ERK1/2 (p-ERK1/2), total ERK1/2 (ERK1/2) and *β*-actin in differentiated-H9c2 cells under oxidative stress conditions that were transfected with scrambled, PLC*δ*1-targeting (#1 and #2), or PLC*δ*3-targeting (#1 and #2) siRNA. (**c**) Immunoblotting of PKC*α*, PKC*β*_1_, PKC*ɛ*, and PKC*θ* in control (*Meox2*^*+/+*^*PLCδ1*^*fl/−*^*PLCδ3*^*+/−*^) or cDKO hearts at 6 weeks of age. Twenty-five micrograms of protein was loaded per lane. GAPDH and Caveolin1 were used as markers for cytosolic and membrane-containing particulate fractions, respectively. (**d**) Immunoblotting of the phosphorylated (active) form of PKC*θ* (^Thr538^p-PKC*θ*) and *β*-actin in differentiated-H9c2 cells that were transfected with scrambled, PLC*δ*1-targeting (#1 and #2), or PLC*δ*3-targeting (#1 and #2) siRNA. (**e**) Immunoblotting of PKC*θ* and *β*-actin in differentiated-H9c2 cells that were transfected with scrambled, or PKC*θ*-targeting (#1 and #2) siRNA. (**f**) Morphologies of differentiated-H9c2 cells with the indicated siRNA. The cells were stained with phalloidin (red) and Hoechst (blue). Scale bar, 100 *μ*m. (**g** and **h**) Population of H9c2 cells with TUNEL-positive nuclei under oxidative stress conditions. Scale bar, 25 *μ*m. Mean+S.E.M. (**i**) Population of H9c2 cells with shrunken nuclei under normal culture conditions in absence or in the presence of 160 nM PMA. Mean+S.E.M. Statistical significance was assessed using Tukey–Kramer's *post hoc* test. *P<0.05 and **P<0.01

Increased apoptosis in the cDKO heart could be caused by impairment of survival signals and activation of proapoptotic signals. Western blotting revealed that activation of Akt was impaired in cDKO hearts and in H9c2 cells under oxidative stress conditions, indicating that the survival signal was impaired by a combined loss of PLC*δ*1 and PLC*δ*3 (Figures 6a and b). In contrast, the combined loss of PLC*δ*1 and PLC*δ*3 did not affect activation of ERK. In addition, the combined loss of PLC*δ*1 and PLC*δ*3 increased the level of activated caspase 9 (Figure 6a), indicating that a proapoptotic signal was activated in the cDKO heart. PKC isozymes have critical roles in the maintenance of cardiac structure and functions. As active PKC isozymes are reported to be present in the particulate fraction of the heart,^21^ we examined the amounts of PKC isozymes in particulate fractions of the heart at 6 weeks of age. The amount of PKC*θ* was decreased in the particulate fraction of the cDKO heart compared with the control heart (Figure 6c and Supplementary Figure S3). In contrast, simultaneous loss of PLC*δ*1 and PLC*δ*3 did not cause remarkable changes in the levels of PKC*α*, PKC*β*_1_, and PKC*ɛ* in the particulate fraction (Figure 6c). Impaired activation of PKC*θ* was also observed in PLC*δ*1/PLC*δ*3 double-knockdown H9c2 cells by examining the amount of phosphorylated PKC*θ* (Figure 6d). Consistent with mild induction of apoptosis by single knockdown of PLC*δ*3 (Figures 5g–j), the amount of phosphorylated PKC*θ* was slightly decreased in PLC*δ*3 single-knockdown H9c2 cells (Figure 6d). These results indicate that simultaneous depletion of PLC*δ*1 and PLC*δ*3 selectively impaired the activation of PKC*θ*. Interestingly, siRNA-mediated silencing of PKC*θ* in H9c2 cells resulted in morphological changes and increased apoptosis, in a manner similar to the combined silencing of PLC*δ*1 and PLC*δ*3 (Figures 6e–h). Furthermore, the numbers of cells with shrunken nuclei were decreased by treating PLC*δ*1/PLC*δ*3 double-knockdown H9c2 cells with a PKC activator, phorbol 12-myristate 13-acetate (PMA) (Figure 6i). These results strongly suggest that PKC*θ* is involved in the morphological changes and high apoptosis caused by the simultaneous knockdown of PLC*δ*1 and PLC*δ*3.

## Discussion

In this study, we demonstrated that the simultaneous loss of PLC*δ*1 and PLC*δ*3 induces cardiac fibrosis, hypertrophy of cardiomyocytes, so-called pathological remodeling, and cardiomyopathy. In addition, we found that double silencing of PLC*δ*1 and PLC*δ*3 resulted in morphological changes in H9c2 cells, which suggests that these enzymes contribute to the maintenance of the shape of cardiomyocytes. Therefore, deletion of these enzymes likely contributes to the induction of cardiomyocyte hypertrophy. Furthermore, we found that the absence of both PLC*δ*1 and PLC*δ*3 causes enlargement of the left ventricular cavity, that is, a DCM-like phenotype. Given that the simultaneous loss of PLC*δ*1 and PLC*δ*3 in the embryo proper did not result in embryonic lethality and that the number of apoptotic cells did not apparently increase in the cDKO embryos, PLC*δ*1 and PLC*δ*3 are specifically required by the adult heart and are dispensable during cardiac development in the embryo.

Cardiomyocyte dropout was often followed by cardiac fibrosis. Predating other cardiac abnormalities, excessive apoptosis was observed in cDKO cardiomyocytes as early as 4 weeks of age, which suggests that excessive apoptosis seems to be a cause for cardiac fibrosis, at least partially. Interestingly, we have reported that simultaneous loss of PLC*δ*1 and PLC*δ*3 results in excessive apoptosis in placental trophoblasts,^22^ which indicates that PLC*δ*1 and PLC*δ*3 regulate cell survival in both cardiomyocytes and trophoblasts. Unexpectedly, the silencing of merely PLC*δ*3 leads to a modest increase in apoptotic cells in H9c2 cells despite no apparent apoptotic phenotypes in the PLC*δ*3 KO heart *in vivo*. Cardiomyocytes may be protected from apoptosis in an *in vivo* environment by unknown mechanisms, and the loss of PLC*δ*3 is insufficient to induce apoptosis.

The mechanisms by which loss of PLC*δ*1 and PLC*δ*3 causes cardiac abnormalities remain to be fully elucidated. Loss of PKC*θ* in mice resulted in cardiac abnormalities, including a reduction in contractile performance, increased end-systolic volume, cardiac fibrosis, hypertrophy of cardiomyocytes, and apoptosis of cardiomyocytes.^21^ All of these cardiac abnormalities were observed in the cDKO mice. In addition, loss of PKC*θ* leads to inhibition of Akt and activation of caspase 9, as does the combined loss of PLC*δ*1 and PLC*δ*3.^21^ PLC*δ*1 and PLC*δ*3 generate diacylglycerol, which is an activator for PKC*θ* and simultaneous depletion of PLC*δ*1 and PLC*δ*3 impaired the activation of PKC*θ*. Therefore, insufficient activation of PKC*θ* is likely to be a cause for cardiac abnormalities in cDKO mice. Accordingly, the silencing of PKC*θ* leads to a partial phenocopy of the combined silencing of PLC*δ*1 and PLC*δ*3 in H9c2 cells. In addition to PKC*θ*, lack of PKC*ɛ* resulted in interstitial fibrosis when the mice were subjected to pressure overload by transverse aortic constriction.^24^ In addition, PKC*ɛ* has a protective role against cardiomyocyte apoptosis during cardiac ischemia/reperfusion injury.^20^ However, simultaneous loss of PLC*δ*1 and PLC*δ*3 did not affect the activation status of PKC*ɛ*. PLC*δ*1 and PLC*δ*3 may specifically regulate the activation of PKC*θ* isozymes by unknown mechanisms. In addition to PKC, PLC activation results in the elevation of the intracellular calcium ion concentration ([Ca^2+^]_i_) and activates calcium-dependent downstream molecules. In cardiomyocytes, cardiac excitation-contraction coupling (ECC) occurs through Ca^2+^-induced Ca^2+^ release (CICR). Although ryanodine receptors are the primary Ca^2+^ release channel that mediates CICR during cardiac ECC, the IP_3_ receptor (IP_3_R) Ca^2+^ release channel is also expressed in cardiomyocytes.^25, 26^ Recent evidence suggests that the activation of IP_3_R may modulate ECC.^27, 28^ Therefore, simultaneous loss of PLC*δ*1 and PLC*δ*3 may result in abnormal Ca^2+^ handling in cardiomyocytes, thereby leading to cardiac abnormalities in cDKO mice. Loss of calcineurin A*β*, which is a calcium-dependent serine-threonine phosphatase, increases the number of apoptotic cardiomyocytes and cardiac fibrosis in mice with DCM.^29, 30^ Given that our cDKO mice showed a DCM-like phenotype, calcineurin A*β* may also be involved in cardiac abnormalities in cDKO mice. In addition to PKC and [Ca^2+^]_i_, the PLC substrate PIP_2_ has roles in cardiac muscle relaxation between contractions by positively regulating a Na^+^/Ca^2+^ exchanger, which removes Ca^2+^ into the extracellular space.^31^ Therefore, impaired hydrolysis of PIP_2_ may also contribute to cardiac abnormalities in cDKO mice.

The findings of this study suggest that PLC*δ*1 and PLC*δ*3 are possible therapeutic targets for DCM and cardiac remodeling that leads to heart failure. Future work will determine whether the expression and/or activity of PLC*δ*1 and PLC*δ*3 in patients with DCM and/or heart failure are decreased.

## Materials and Methods

### Mice

*PLCδ3*^*−/−*^ mice and *PLCδ1*^*fl/fl*^ mice (Acc. No. CDB0552K: http://www.cdb.riken.jp/arg/mutant%20mice%20list.html) were produced as described previously.^13, 22^ *Meox2*-*Cre* mice were obtained from the Jackson Laboratory. The genotyping primers for *PLCδ1* were 5′-CTGGGATCTTGGTGGGCATCG-3′, 5′-CCACACCGAGCCAAGCTCAC-3′, and 5′-CCTGTGCTCTAGTAGCTTTACG-3′. The genotyping primers for *PLCδ3* were 5′-TTAACCTGATGCTCCTGAGG-3′, 5′-GGATAAAATGCTTGCCCTGC-3′, and 5′-AAAATGGCGTTACTTAAGCTAGCTT-3′. The genotyping primers for the *PLCδ1flox* allele were 5′-GAATGTGACCACTCCCAGAC-3′, and 5′-CAGGAGGAAGATGAGGCCCA-3′. The genotyping primers for *Meox2-Cre* were 5′-GGGACCACCTTCTTTTGGCTTC-3′, 5′-AAGATGTGGAGAGTTCGGGGTAG-3′, and 5′-CCAGATCCTCCTCAGAAATCAGC-3′. All the animal studies were approved by the animal experiments review board of the Tokyo University of Pharmacy and Life Sciences.

### Real-time RT-PCR

Total RNA was isolated using an RNeasy Mini kit (Qiagen, Hilden, Germany) according to the manufacturer's protocol. Template complementary DNA was synthesized from total RNA by using a ReverTra Ace qPCR RT kit (Toyobo, Osaka, Japan). Real-time PCR was performed using THUNDERBIRD SYBR qPCR Mix (Toyobo) in a CFX96 thermocycler (Bio-Rad, München, Germany). The primer sequences are listed in Table 2. The relative amounts of mRNA were normalized to glyceraldehyde-3-phosphate dehydrogenase (GAPDH) mRNA levels.

### Histochemistry

For histological analyses, placenta or hearts were fixed in 4% paraformaldehyde, dehydrated, and embedded in paraffin. Sections (5 *μ*m thick) were stained with HE. Trichrome staining was performed with a Trichrome Stain (Masson) Kit (Sigma, St Louis, MO, USA) according to the manufacturer's instructions. Sections were examined under a BX51 microscope (Olympus, Tokyo, Japan).

### TUNEL assay

TUNEL assay was performed on paraffin sections with an *In Situ* Cell Death Detection Kit, TMRred (Roche, Basel, Switzerland). Counter-staining was performed with Hoechst 33258 (Life Technologies, Carlsbad, CA, USA). Sections were observed under a BZ-8000 microscope (Keyence, Osaka, Japan).

### Culturing and siRNA-mediated gene silencing of H9c2 cells

The H9c2 rat cardiomyoblast cell line was purchased from DS Pharma Biomedical (Osaka, Japan). The cells were maintained in a proliferative state by culturing them in Dulbecco's modified Eagle's medium (DMEM) that was supplemented with 10% fetal bovine serum at 37 °C in a humidified atmosphere with 5% CO_2_. siRNA against rat PLC*δ*1, rat PLC*δ*3, and rat PKC*θ* was purchased from Invitrogen (Stealth RNAi) and transfected with Lipofectamine RNAiMAX (Life Technologies) at 40 nM. Two individual non-overlapping Stealth RNAi duplexes per target were used for all experiments and closely similar results were obtained with these Stealth RNAi duplexes. Differentiation into cardiomyocytes was induced by changing the medium to DMEM with 1% FBS for 72 h. For PMA treatment, H9c2 cells were treated with 160 nM PMA for 48 h. In some experiments, H9c2 cells were treated with 100 *μ*M H_2_O_2_ for 1 h. After a further 24 h of culturing with DMEM with 1% FBS, cell death was determined by staining with Hoechst 33342 (DOJINDO, Japan) or by using an *In Situ* Cell Death Detection Kit, TMRred (Roche). The cells were observed under a BZ-8000 microscope (Keyence).

### Echocardiography

Echocardiography was performed as described previously.^32^ Mice were anesthetized with an intraperitoneal injection of 25 mg/kg pentobarbital (Sigma). After anesthesia, the left hemithorax of each mouse was shaved. The animals were pre-warmed with a panel heater to maintain their rectal temperature at 37 °C during the determination of cardiac parameters by echocardiography. Transthoracic echocardiography was performed using ProSound 5500R (Aloka, Tokyo, Japan) with a 13-MHz linear transducer for mice in a phased array format, which offered a lateral resolution of 0.35 mm and an axial resolution of 0.25 mm, real-time digital acquisition, storage, and review capabilities. Each cardiac parameter was calculated from the echocardiogram as described previously.^32^

### Measurement of blood pressure

Systolic blood pressure of nonanesthetized mice was measured by tail-cuff blood pressure measurement using a computerized CODA high-throughput noninvasive BP acquisition system (Kent Scientific Corp., Torrington, CT, USA), in accordance with the manufacturer's instructions. Briefly, the mice were placed in a warmed chamber. Cuffs were placed around the mouse tail to measure arterial systolic pressure. Three to five readings were recorded for each animal. Measurements were repeated in the event of animal movement or weak pressure/flow recordings.

### Preparation of subcellular protein fractions from hearts

Preparation of subcellular protein fractions from hearts was performed as described.^21^ Briefly, hearts were homogenized in homogenization buffer (20 mM Tris at pH 7.5, 2 mM EGTA, 2 mM EDTA, 250 mM sucrose, 5 mM DTT, and Complete Protease Inhibitor Cocktail tablets (Roche)) and incubated for 30 min on ice. The samples were then spun at 100 000 × *g* for 30 min at 4 °C. The supernatant was stored as the cytosolic fraction, whereas the remaining pellet was further suspended in homogenization buffer containing 0.1% Triton X-100 and incubated for 30 min on ice. Then, the samples were spun at 100 000 × *g* for 30 min at 4 °C and the remaining supernatant was stored as the particulate fraction. An equal amount of each sample was subjected to immunoblotting.

### Antibodies

The following antibodies were used for immunoblotting: PLC*δ*1, PLC*δ*3 (lab-made); *β*-actin, Tubulin (SIGMA); PKC*α* (BD Biosciences, San Jose, CA, USA); PKC*β*_1_, PKC*θ*, Caveolin1, Bax (Santa Cruz Biotechnology Inc., Santa Cruz, CA, USA); and PKC*ɛ*, ^Thr538^p-PKC*θ*, Bad, Bcl-2, Akt, ^Ser473^p-Akt, ERK, p-ERK, cleaved Caspase 9, Glyceraldehyde-3-phosphate dehydrogenase (GAPDH) (Cell Signaling Technology Inc., Danver, MA, USA).

## Figures and Tables

**Table 1:** Genotyping of offspring from Meox2^+/+^PLC*δ*1^fl/fl^PLC*δ*3^*−*/*−*^ × Meox2^cre/+^PLC*δ*1^*−*/*−*^PLC*δ*3^+/*−*^ mating

	***Meox2***^***+/+***^		***Meox2***^***cre/+***^		
	***δ1***^***fl/**−*^***δ3***^***+/**−*^	***δ1***^***fl/**−*^***δ3***^*−**/**−*^	***δ1***^***fl/**−*^***δ3***^***+/**−*^	***δ1***^***fl/**−*^***δ3***^*−**/**−*^	**Total**
No. with genotype	70 (64)	64 (64)	62 (64)	62 (64)	258

The numbers in parentheses were calculated from the expected Mendelian frequencies

**Table 2:** Real-time reverse-transcription PCR primers

ANP	5′-TCGTCTTGGCCTTTTGGCT-3′
	5′-TCCAGGTGGTCTAGCAGGTTCT-3′
BNP	5′-AAGTCCTAGCCAGTCTCCAGA-3′
	5′-GAGCTGTCTCTGGGCCATTTC-3′
*α*-MHC	5′-CTCAGCCAGGCCAATAGAAT-3′
	5′-GACTCCATCTTCTTCTTCTGG-3′
*β*-MHC	5′-ATGTGCCGGACCTTGGAAG-3′
	5′-CCTCGGGTTAGCTGAGAGATCA-3′
TGF-*β*2	5′-AGAGCTCGAGGCGAGATTTG-3′
	5′-TTCTGATCACCACTGGCATATGT-3′
TGF-*β*3	5′-CAATCCGTTCTTTCTGCAACTG-3′
	5′-CTAATTACATGATCTGTGATGACTCATGA-3′
CTGF	5′-CTTCTGCGATTTCGGCTCC-3′
	5′-TACACCGACCCACCGAAGA-3′
Fibronectin1	5′-AAGGTTCGGGAAGAGGTTGT-3′
	5′-CCGTGTAAGGGTCAAAGCAT-3′
Col1A1	5′-AAACCCGAGGTATGCTTGATCTGTA-3′
	5′-GTCCCTCGACTCCTACATCTTCTGA-3′
Col3A1	5′-TGAATGGTGGTTTTCAGTTCAG-3′
	5′-GATCCCATCAGCTTCAGAGACT-3′
MMP-2	5′-CCCCATGAAGCCTTGTTTACC-3′
	5′-TTGTAGGAGGTGCCCTGGAA-3′
TIMP-1	5′-GCCTACACCCCAGTCATGGA-3′
	5′-GGCCCGTGATGAGAAACTCTT-3′
Bax	5′-AAAGTGCCCGAGCTGATCA-3′
	5′-AGCCACAAAGATGGTCACTGTCT-3′
GAPDH	5′-CCATGCCATCACTGCCACCC-3′
	5′-TGTCATCATACTTGGCAGGTTTC-3′

## Abbreviations

PLC: phospholipase C
PKC: protein kinase C
siRNA: small interfering RNA
cDKO: conditional double knockout
DCM: dilated cardiomyopathy
PIP_2_: phosphatidylinositol 4,5-bisphosphate
IP_3_: 1,4,5-trisphosphate
E: embryonic day
EF: ejection fraction
FS: fractional shortening
TUNEL: terminal transferase dUTP nick end labeling
PMA: phorbol 12-myristate 13-acetate
