# Supplementary figures and images for: Simultaneous loss of phospholipase Cδ1 and phospholipase Cδ3 causes cardiomyocyte apoptosis and cardiomyopathy
Source: Cell Death Dis. 2014 May 8;5(5):e1215–. doi: 10.1038/cddis.2014.181 (PMC4047916; doi:10.1038/cddis.2014.181)

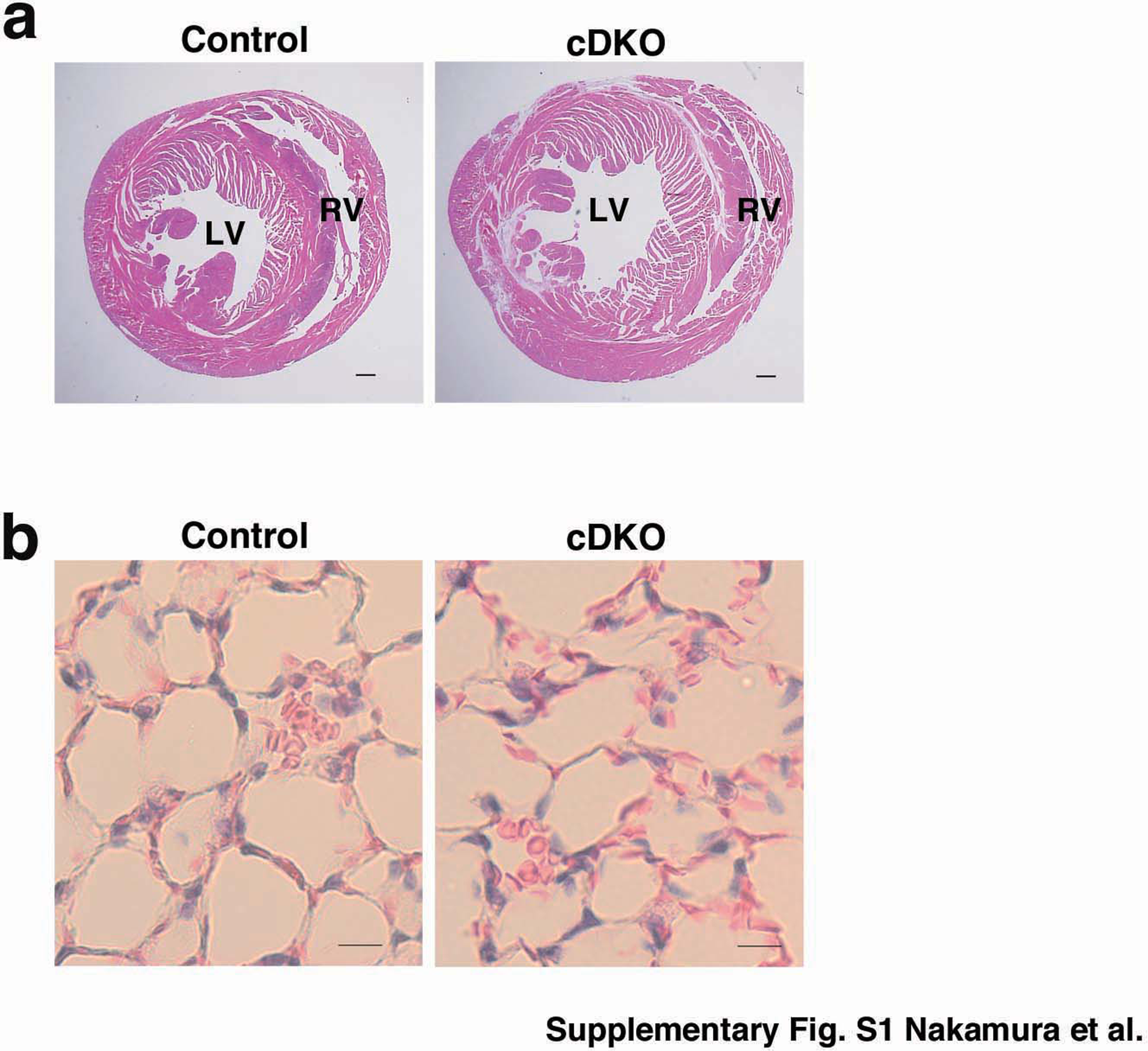

Supplement: Supplementary Figure S1 [file cddis2014181x1.tif]

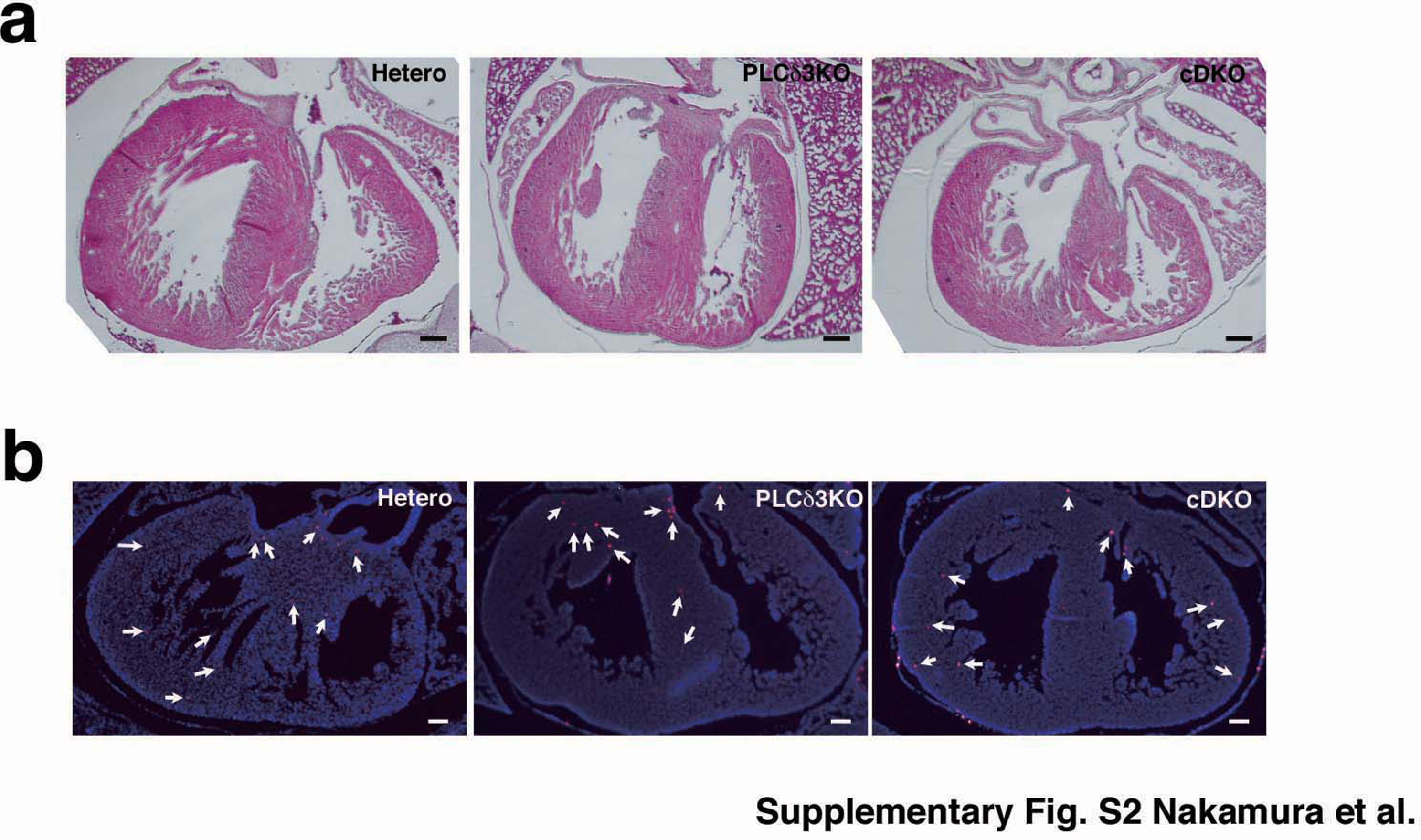

Supplement: Supplementary Figure S2 [file cddis2014181x2.tif]

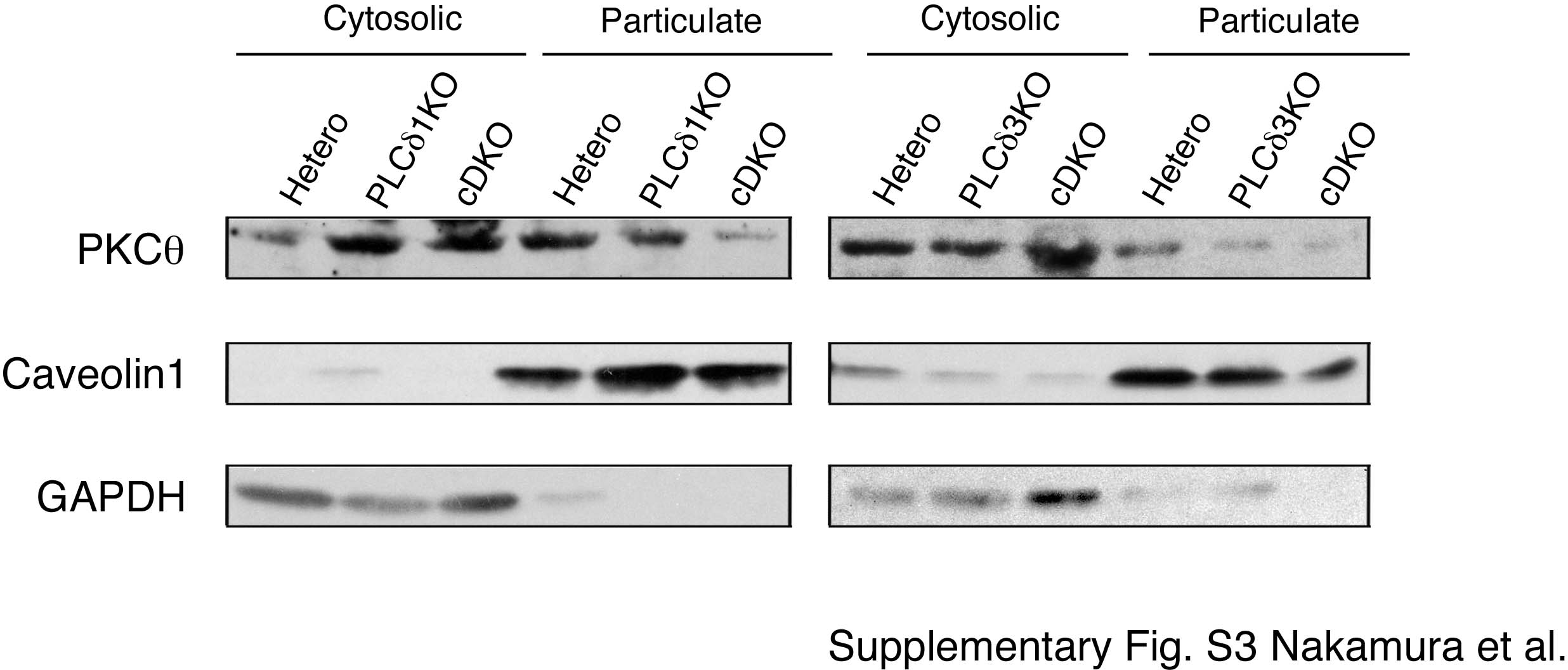

Supplement: Supplementary Figure S3 [file cddis2014181x3.tif]
