# Supplementary material for: Simultaneous loss of phospholipase Cδ1 and phospholipase Cδ3 causes cardiomyocyte apoptosis and cardiomyopathy
Source: Cell Death Dis. 2014 May 8;5(5):e1215–. doi: 10.1038/cddis.2014.181 (PMC4047916; doi:10.1038/cddis.2014.181)
Supplement: Supplementary Figure Legends [file cddis2014181x4.doc]

**Supplementary Figure S1.** cDKO mice did not show lung and right ventricle abnormalities. **(a)** Hematoxylin and eosin -stained sections of control (*Meox2^+/+^PLCδ1^fl/-^PLCδ3^+/-^*) and cDKO hearts at 12 weeks of age. Scale bar, 300 μm. LV, left ventricle; RV, right ventricle. **(b)** Hematoxylin and eosin -stained sections of control (*Meox2^+/+^PLCδ1^fl/-^PLCδ3^+/-^*) and cDKO lungs at 12 weeks of age. Scale bar, 10 μm.

**Supplementary Figure S2.** Apparent cardiac abnormalities were not observed in cDKO embryo at embryonic day (E) 17.5. **(a)** Hematoxylin and eosin -stained sections of *Meox2^+/+^PLCδ1^fl/-^PLCδ3^+/-^* (Hetero), *Meox2^+/+^PLCδ1^fl/-^PLCδ3^-/-^* (PLCδ3KO), and *Meox2^cre/+^PLCδ1^fl/-^PLCδ3^-/-^* (cDKO) hearts at E17.5. Scale bar, 200 μm. **(b)** TUNEL staining (red) of hearts from E17.5 *Meox2^+/+^PLCδ1^fl/-^PLCδ3^+/-^* (Hetero), *Meox2^+/+^PLCδ1^fl/-^PLCδ3^-/-^* (PLCδ3KO), and *Meox2^cre/+^PLCδ1^fl/-^PLCδ3^-/-^* (cDKO) mice. Hoechst (blue) was used for nuclear staining. Scale bar, 100 μm.

**Supplementary Figure S3.** PKCθ activation was impaired in cDKO heart. Immunoblotting of PKCθ in *Meox2^+/+^PLCδ1^fl/-^PLCδ3^+/-^* (Hetero), *Meox2^cre/+^PLC^fl/-^PLCδ3^+/-^* (PLCδ1KO), *Meox2^+/+^PLCδ1^fl/-^PLCδ3^-/-^* (PLCδ3KO), and *Meox2^cre/+^PLCδ1^fl/-^PLCδ3^-/-^* (cDKO) hearts at 8 weeks of age. Twenty-five micrograms of protein was loaded per lane. GAPDH and Caveolin1 were used as markers for cytosolic and membrane-containing particulate fractions, respectively. 2 distinct littermates were used for the experiment.
