# Supplementary material for: Simultaneous loss of phospholipase Cδ1 and phospholipase Cδ3 causes cardiomyocyte apoptosis and cardiomyopathy
Source: Cell Death Dis. 2014 May 8;5(5):e1215–. doi: 10.1038/cddis.2014.181 (PMC4047916; doi:10.1038/cddis.2014.181)
Supplement: Supplementary Tables [file cddis2014181x5.doc]

|  | *Hetero*  *(n=6)* | *PLCδ1KO*  *(n=3)* | *PLCδ3KO*  *(n=3)* | *cDKO*  *(n=4)* |
| --- | --- | --- | --- | --- |
| Fibronectin | 0.75 ± 0.076 | 1.3 ± 0.066 | 1.2 ± 0.097 | 7.5 ± 3.1 |
| Col1A1 | 0.80 ± 0.081 | 1.1 ± 0.23 | 1.3 ± 0.13 | 4.9 ± 1.5 |
| Col3A1 | 0.82 ± 0.063 | 1.1 ± 0.11 | 1.3 ± 0.018 | 4.4 ± 1.0 |
| CTGF | 0.87 ± 0.12 | 1.2 ± 0.21 | 1.2 ± 0.11 | 3.4 ± 0.40 |
| TGFβ2 | 0.94 ± 0.054 | 1.1 ± 0.087 | 1.1 ± 0.075 | 2.3 ± 0.18 |
| TGFβ3 | 0.93 ± 0.053 | 0.99 ± 0.10 | 1.1 ± 0.061 | 2.8 ± 0.30 |
| TIMP-1 | 0.95 ± 0.084 | 0.93 ± 0.22 | 1.2 ± 0.15 | 1.7 ± 0.16 |
| MMP-2 | 0.86 ± 0.11 | 1.0 ± 0.16 | 1.3 ± 0.12 | 2.5 ± 0.29 |

Supplementary Table S1 Relative expression of markers for cardiac fibrosis and remodeling in hearts of 8 weeks-old mice

Mean ± s.e.m.

Hetero; *Meox2^+/+^PLCδ1^fl/-^PLCδ3^+/-^*,

PLCδ1KO *; Meox2^cre/+^PLC^fl/-^PLCδ3^+/-^*,

PLCδ3KO *; Meox2^+/+^PLCδ1^fl/-^PLCδ3^-/-^*,

cDKO; *Meox2^cre/+^PLCδ1^fl/-^PLCδ3^-/-^*.

| 4 weeks  (Male) | *Hetero*  *(n=3)* | *PLCδ1KO*  *(n=3)* | *PLCδ3KO*  *(n=3)* | *cDKO*  *(n=4)* |
| --- | --- | --- | --- | --- |
| Systolic (mmHg) | 100 ± 2.4 | 110 ± 1.5 | 110 ± 2.1 | 100 ± 2.3 |
| Diastolic (mmHg) | 82 ± 1.1 | 92 ± 0.69 | 88 ± 1.8 | 79 ± 1.6 |
| Mean (mmHg) | 89 ± 0.91 | 98 ± 0.84 | 97 ± 1.6 | 87 ± 1.7 |
| 4 weeks | *Hetero*  *(n=3)* | *PLCδ1KO*  *(n=3)* | *PLCδ3KO*  *(n=3)* | *cDKO*  *(n=3)* |
| Systolic (mmHg) | 110 ± 4.6 | 100 ± 1.0 | 110 ± 0.95 | 110 ± 3.6 |
| Diastolic (mmHg) | 86 ± 4.0 | 82 ± 1.4 | 85 ± 1.9 | 87 ± 2.9 |
| Mean (mmHg) | 95 ± 4.1 | 89 ± 1.2 | 93 ± 1.4 | 94 ± 3.1 |
| 6 weeks (Male) | *Hetero*  *(n=3)* | *PLCδ1KO*  *(n=4)* | *PLCδ3KO*  *(n=3)* | *cDKO*  *(n=4)* |
| Systolic (mmHg) | 120 ± 3.7 | 110 ± 1.5 | 110 ± 2.5 | 120 ± 3.9 |
| Diastolic (mmHg) | 85 ± 3.9 | 92 ± 0.77 | 83 ± 1.7 | 91 ± 1.8 |
| Mean (mmHg) | 97 ± 3.7 | 99 ± 0.89 | 93 ± 1.8 | 100 ± 2.3 |
| 6 weeks (Female) | *Hetero*  *(n=3)* | *PLCδ1KO*  *(n=4)* | *PLCδ3KO*  *(n=3)* | *cDKO*  *(n=4)* |
| Systolic (mmHg) | 120 ± 3.1 | 110 ± 2.9 | 120 ± 3.9 | 110 ± 2.0 |
| Diastolic (mmHg) | 85 ± 2.1 | 87 ± 2.0 | 85 ± 2.0 | 89 ± 1.1 |
| Mean (mmHg) | 96 ± 2.2 | 96 ± 2.0 | 97 ± 2.3 | 96 ± 1.4 |

Supplementary Table S2 Systolic, diastolic, and mean blood pressures of 4 or 6 weeks-old mice

Mean ± s.e.m.

Hetero; *Meox2^+/+^PLCδ1^fl/-^PLCδ3^+/-^*,

PLCδ1KO *; Meox2^cre/+^PLC^fl/-^PLCδ3^+/-^*,

PLCδ3KO *; Meox2^+/+^PLCδ1^fl/-^PLCδ3^-/-^*,

cDKO; *Meox2^cre/+^PLCδ1^fl/-^PLCδ3^-/-^*.
